# Airway Mucins Inhibit Oxidative and Non-Oxidative Bacterial Killing by Human Neutrophils

**DOI:** 10.3389/fphar.2020.554353

**Published:** 2020-09-30

**Authors:** André M. Cantin, Cristine Ouellet, Alexandre Cloutier, Patrick P. McDonald

**Affiliations:** Pulmonary Division, Faculty of Medicine and Health Sciences, Université de Sherbrooke, Sherbrooke, QC, Canada

**Keywords:** cationic peptides, NADPH oxidase, *Pseudomonas aeruginosa*, cystic fibrosis, NADH oxidase, *Burkholderia cepacia*

## Abstract

Neutrophil killing of bacteria is mediated by oxidative and non-oxidative mechanisms. Oxidants are generated through the NADPH oxidase complex, whereas antimicrobial proteins and peptides rank amongst non-oxidative host defenses. Mucus hypersecretion, deficient hydration and poor clearance from the airways are prominent features of cystic fibrosis (CF) lung disease. CF airways are commonly infected by *Pseudomonas aeruginosa* and *Burkholderia cepacia* *complex* bacteria. Whereas the former bacterium is highly sensitive to non-oxidative killing, the latter is only killed if the oxidative burst is intact. Despite an abundance of neutrophils, both pathogens thrive in CF airway secretions. In this study, we report that secreted mucins protect these CF pathogens against host defenses. Mucins were purified from CF sputum and from the saliva of healthy volunteers. Whereas mucins did not alter the phagocytosis of *Pseudomonas aeruginosa* and *Burkholderia cenocepacia* by neutrophils, they completely suppressed bacterial killing. Accordingly, mucins markedly inhibited non-oxidative bacterial killing by neutrophil granule extracts, or by lysozyme and the cationic peptide, human β defensin-2 (HBD2). Mucins also suppressed the neutrophil oxidative burst through a charge-dependent mechanism that could be reversed by the cationic aminoglycoside, tobramycin. Our data indicate that airway mucins protect Gram-negative bacteria against neutrophil killing by suppressing the oxidative burst and inhibiting the bactericidal capacity of cationic proteins and peptides. Mucin hypersecretion, dehydration, stasis and anionic charge represent key therapeutic targets for improving host defenses and airway inflammation in CF and other muco-secretory airway diseases.

## Introduction

Cystic fibrosis (CF) is a fatal autosomal recessive disease that limits to 50 years or less the median age of patients’ survival (Elborn, 2016). Almost all of the mortality associated with CF stems from respiratory insufficiency accompanied by repeated airway infections (Lund-Palau et al., 2016). Airway infections with *Pseudomonas aeruginosa* and infectious respiratory exacerbations (Waters et al., 2012) predict poor lung health and mortality in children and adults with CF (Emerson et al., 2002). The two cardinal features of CF lung disease are chronic infection and excessive inflammation (Boucher, 2002). The lack of functional cystic fibrosis transmembrane conductance regulator protein (CFTR) is associated with deficient cAMP-dependent chloride secretion into the CF airway lumen (Boucher, 2007). Consequently, airway mucus is dehydrated, mucins are concentrated and mucociliary clearance is deficient (Perez-Vilar and Boucher, 2004). Persistent bronchopulmonary infections ensue, leading to tissue destruction and respiratory insufficiency (Bergeron and Cantin, 2019).

Neutrophils play a major role in lung host defense. Almost all neutropenic mice die within 7 days of intratracheal instillation of a small number (10^4^ CFU) of *P. aeruginosa* bacteria (Oishi et al., 1993). Both non-oxidative and oxidative mechanisms of killing bacteria are present in airway secretions and neutrophils. The reactive oxygen species (ROS) generated by the oxidative burst are essential for the antimicrobial functions of neutrophils as evidenced by life-threatening infections that afflict individuals with chronic granulomatous disease (CGD), a disease characterized by genetically determined deficiencies in NADPH oxidase function (Dinauer, 2007; Rieber et al., 2012). The spectrum of pathogens infecting individuals with CF is similar to that observed in CGD, comprising bacteria and fungi that cannot produce hydrogen peroxide. Furthermore, *B. cepacia* complex (Bcc) is a rare group of pathogens in humans that is observed almost exclusively in CF and CGD, suggesting that there may be similarities in the host defense defects of both diseases. Interestingly, neutrophils isolated from CF sputum, but not from CF blood, have a deficient oxidative burst and the degree of this deficiency correlates with the severity of lung function impairment (Houston et al., 2013). Furthermore, low concentrations of chloramines, neutrophil-derived oxidants, in airway secretions is associated with worse lung function in CF patients (Witko-Sarsat et al., 1995).

Neutrophil-derived oxidants are produced by NADPH oxidase. NADPH oxidase function depends upon the transfer of electrons to the extracellular milieu or to the phagosome, where oxygen is reduced to superoxide. The movement of electrons during the oxidative burst results in a current that favors depolarization of the neutrophil plasma membrane. NADPH oxidase function is preserved over a wide range of transmembrane voltages (-190 to 0 mV), but decreases rapidly above 0 mV, and is abolished at +190 mV (DeCoursey et al., 2003). Excessive depolarization of the neutrophil membrane during the oxidative burst inhibits NADPH oxidase unless H^+^ simultaneously leaves the cell through a voltage-gated proton channel and limits the degree of depolarization (Henderson et al., 1987). High molecular weight glycosaminoglycans can contribute to the depolarization of cell membrane potential (Hagenfeld et al., 2010).

MUC5AC and MUC5B, the major mucins in airway secretions, are polymeric glycoproteins (Evans and Koo, 2009) bearing important negative charges, particularly in CF (Schulz et al., 2007). Secreted mucins are the most abundant component of CF sputum (Thornton et al., 1991; Thornton et al., 2008). We hypothesized that the concentration of anionic mucins in the CF airway creates an environment that could induce neutrophil membrane depolarization during the oxidative burst and inhibit NADPH oxidase activity. Furthermore, we hypothesized that the anionic charge of mucins may also inhibit non-oxidative bacterial killing by cationic proteins and peptides.

## Methods

### Human Neutrophil Isolation

Neutrophils were isolated from the peripheral blood of healthy donors, following a protocol that was approved by an institutional ethics committee (Comité d’éthique de la recherche du CIUSS de l’Estrie-CHUS). The entire procedure was carried out at room temperature and under endotoxin-free conditions, as described previously (Ear et al., 2005). Purified neutrophils were resuspended in RPMI 1640 supplemented with 5% autologous serum, at a final concentration of 5 × 106 cells/ml (unless otherwise stated). As determined by Wright staining and FACS analysis, the final neutrophil suspensions contained fewer than 0.1% monocytes or lymphocytes; neutrophil viability exceeded 98% after up to 4 h in culture, as determined by trypan blue exclusion and by Annexin V/propidium iodide FACS analysis.

### Zymosan Preparation

Zymosan (Sigma-Aldrich) was suspended in 0.9% saline at 12 mg/ml, heated at 100°C for 1 h, centrifuged, and washed in 0.9% saline. It was then suspended in Krebs-Ringer phosphate buffer pH 7.35 supplemented with 2 mg/ml dextrose, at a concentration of 50 mg/ml. Fresh human serum was then added (1:3, v/v) and the mixture was incubated for 20 min at 37°C with gentle agitation. This results in the opsonization of zymosan, both by immunoglobulins and complement components (Cheson and Morris, 1981). The opsonized zymosan was then centrifuged (8,000 × g, 15 min), washed with DMEM, and suspended in DMEM at a final concentration of 10 mg/ml.

### Neutrophil Granule Extraction

Neutrophils from healthy volunteers were suspended and lysed in 0.2 M sucrose solution (containing 1 mg/ml of heparin sodium and 5 µg/ml of DNAse) overnight at 4°C under agitation. The mixture was then centrifuged (1000 × g, 10 min, 4°C), and the resulting supernatant was further centrifuged (30,000 × g, 45 min, 4°C). The granule pellet was resuspended in 500 µl of 0.05 M sodium acetate and 1 M NaCl (pH 4.0). Granules were disrupted in a Potter-Elvehjem homogenizer and centrifuged (20,000 × g, 15 min, 4°C). The supernatant was collected and the pH adjusted to 7.4 with 3 M TrisBase (Baugh and Travis, 1976). The total protein content of neutrophil granule extracts was quantified using the Bio-Rad Protein Assay Kit (Bio-Rad Laboratories, Hercules CA).

### Purification and Characterization of Mucins

Sputum was collected from 2 CF adult individuals (1 male, 25 years old, FEV1 of 40%, BMI of 21.7; 1 female, 29 years old, FEV1 of 86%, BMI of 21.5, both non-smokers) pooled, and stored at -80°C. CF adults spontaneously produced more than 10 g of sputum per 20-min session of chest physiotherapy. Each subject had a CF diagnosis based on usual clinical criteria and each was homozygous for the F508del mutation. Both CF individuals were in stable condition without respiratory exacerbation at the time of sputum collection. Salivary samples were also collected from each CF participant and pooled or collected from asymptomatic healthy volunteers (n = 12, 4 male, 9 female; mean age, 33.4 ± 4.0; all non-smokers), pooled, and stored at -80°C.

For mucin purification, each sputum sample (CF airway, CF saliva, and healthy volunteer saliva) was liquefied with a buffered solution containing 0.2 M NaCl, 10 mM EDTA, 2 mM Pefabloc, and 1 mg/ml of DNAse (pH 7.9) for 1 h at 37°C and a further 120 min at RT with gentle agitation prior to centrifugation in a Sorvall Superspeed RC 2-B (27,000 × g, 20 min, 4°C). The supernatant was collected and passed through a PD-10 column in PBS supplemented with 1 M NaCl and deposited on a Sepharose 4B (Sigma-Aldrich, Oakville, ON, Canada) column in PBS with 1 M NaCl. Elution fractions were monitored at 280 nm (Beckman DU-7 spectrophotometer), and the first eluted fraction was collected and deposited on a strong cation exchange Sepharose^®^ media (Hitrap SP, Amersham Biosciences) in PBS at pH 7. The fraction that did not bind was collected and dialyzed (Spectra/por membrane 12,000–14,000 MW, Spectrum Laboratories, Rancho Dominguez, CA) in water for 72 h at 4°C with agitation, lyophilizied, and sterilized in a gamma cell (Gammacell 220, Nordion, Canada).

Saliva was collected from CF volunteers or healthy volunteers and gently mixed with a magnetic stirring bar in an equal volume of 0.1 M NaCl at 4°C overnight. The sample was then centrifuged (4,400 × g, 30 min, 4°C), and the resulting supernatant was collected and adjusted to pH 7.6 with 500 mM Na_2_HPO_4_. Subsequent steps were those described above for CFAM (Raynal et al., 2002; Raynal et al., 2003).

#### Western Blot Analysis on Agarose Gel

Samples of CFAM (200 μg) and salivary mucin from healthy volunteers (200 μg) were electrophoresed on a 0.7% agarose gel. The separated proteins were transferred to a nitrocellulose membrane (0.45 μm; Bio-Rad Laboratories Ltd., Mississauga, ON, Canada) using the Vacuum Blotter system (Model 785, Bio-Rad Laboratories Ltd., Mississauga, ON, Canada) for 2 h and processed for western blotting, using 5% (w/v) milk as the blocking agent in TBS (pH 7.6) for 1 h at RT. The membranes were then incubated with goat polyclonal MUC5B IgG (1:250, Santa Cruz Biotechnology, Santa Cruz, CA) or MUC5AC-Concentrated-Clone B442 (1:250, Biomeda, Foster City, CA) overnight at 4°C and washed three times in TBS. Membranes were incubated with swine anti-goat IgG peroxidase (1:5,000, Cedarlane, Burlington, ON, Canada) for 1 h at RT and revealed by chemiluminescence (ECL Kit; Amersham, Buckinghamshire, UK) (Kirkham et al., 2002).

### Bacterial Cultures

Stocks of *P. aeruginosa* (PAO1, American Type Culture Collection, Manassas, VA) and *Burkholderia cenocepacia* (C5424, ET 12 lineage from sputum of a CF patient, provided by Dr. David Speert, University of British Columbia) were maintained at -80°C in 10% glycerol and plated on MacConkey agar (Becton Dickinson, Sparks, MD) at 37°C for 18 h. A single colony of PAO1 on MacConkey agar was incubated in 15 ml of Müller-Hinton broth overnight at 37°C with agitation. Either 20 μl of *B. cenocepacia* in 10% glycerol or two to three single colonies on agar were incubated in 3 ml of Trypticase Soy medium (Becton, Dickinson and Company) and incubated overnight at 37°C with agitation. The absorbance was determined in a spectrophotometer at 600 nm and adjusted to 0.1 absorbance units × cm^-1^ (AU/cm^-1^) for *P. aeruginosa* and 0.2 (AU/cm^-1^) for *B. cenocepacia* in 10 ml of fresh medium. The subcultures were incubated at 37°C (2–3 h), grown to exponential phase (0.4–0.5 AU/cm^-1^), and adjusted to obtain the desired bacterial concentrations (0.5 AU/cm^-1^ = 400 × 10^6^ P*. aeruginosa*/ml, 0.5 AU/cm^-1^ = 600 × 10^6^ *B. cenocepacia*/ml).

### Neutrophil-Mediated Bacterial Killing

The subcultures of *P. aeruginosa* or *B. cenocepacia* and human neutrophils were adjusted, respectively, to 2.5 × 10^5^ bacteria/ml and 5 × 10^5^ neutrophils/ml in HBSS containing calcium, magnesium, and 1% human serum. Bacteria and CFAM (1, 2, or 4 mg/ml) or salivary mucins (1, 2, or 4 mg/ml) were incubated 20 min at RT. Bacteria, CFAM or salivary mucins, and human neutrophils were mixed and incubated for 2 h at 37°C with gentle agitation. After incubation, these suspensions were diluted in water for 10 min at RT for cell lysis and vortexed for 30 s, and serial dilutions were spread on Müller-Hinton agar plates and incubated overnight at 34°C. To determine the effect of CFAM and salivary mucins on neutrophil-mediated bacterial killing, colony-forming units (CFU) were counted by standard plate-counting procedures (Vishwanath et al., 1988).

### Phagocytosis Assays and ^125^I Mucin Uptake

Subcultures of *P. aeruginosa* or *B. cenocepacia* (50 × 10^6^ bacteria/ml) were incubated in HBSS containing calcium, magnesium, and 5% autologous human serum for 10 min at RT before the addition of neutrophils (5 × 10^6^ cells/ml). Bacteria, neutrophils and CFAM or salivary mucins were mixed and incubated 1 h at 37°C with gentle agitation. After incubation, reactions were stopped with HBSS at 4°C (containing 10% serum, without calcium and magnesium). To determine the number of bacteria associated with each neutrophil, cytospin preparations (50,000–100,000 neutrophils/slide) were stained (Hemacolor Kit, EM Science, Gibbstown), and bacteria were counted in 50 neutrophils per slide (Vishwanath et al., 1988).

CFAM was labeled with ^125^I in the presence of chloramine-T (Sigma-Aldrich) (Hunter and Greenwood, 1962). ^125^I-labeled CFAM (70 µg/ml, 1.43 × 10^6^ cpm/ml) was added to cold CFAM to achieve a final concentration of 4 mg/ml. Bacteria, neutrophils, and CFAM were mixed and incubated 1 h at 37°C or at 4°C, with gentle agitation. Cells were then centrifuged in HBSS (300 × g, 10 min, 4°C) and washed in 30 volumes of HBSS at 4°C. Radioactivity was then counted in the cell pellets.

### Neutrophil Oxidative Burst

Neutrophil oxidative burst was determined by measuring luminol-amplified chemiluminescence with the membrane-permeable dye, luminol (5-amino-2,3-dihydro-1,4-phtalazinedione; Sigma-Aldrich), as described by (Dahlgren et al. (2007). In an adapted 96-well microtiter plate, a mixture was prepared, consisting in 100 µl of HBSS buffer, 50 U/ml of superoxide dismutase, 200 U/ml of catalase, and 10^7^ PAO1, 5 mg/ml of opsonized, zymosan, or 1 μg/ml of tetradecanoyl phorbol acetate (PMA) in the presence of 0–2 mg/ml of CFAM or salivary mucins. The oxidative burst was initiated by adding 100 µl HBSS containing 5 × 10^6^/ml neutrophils and 50 µM luminol. Chemiluminescence was detected in a Fusion Packard chemiluminometer (PerkinElmer, Inc., Waltham, MA). In addition to luminol-amplified chemiluminescence, neutrophil superoxide production was measured by ferricytochrome *c* reduction. Neutrophils were suspended in HBSS containing 80 µM ferricytochrome *c* (Sigma) and incubated for 30 min at 37°C. The absorbance of supernatants was assessed at a wavelength of 550 nm. The effect of mucin charge neutralization was determined by adding 1–4 mg/ml of tobramycin, a cationic aminoglycoside, to the neutrophils in the presence of 2 mg/ml of CFAM. Neutrophils were stimulated with opsonized zymosan and superoxide was measured by the reduction of ferricytochrome *c*.

### Membrane Depolarization Assays Using Flow Cytometry

Human neutrophils (5 × 10^6^/ml in HBSS) were incubated with respiratory mucin, PMA or the combination of mucin and PMA for 20 min at 37°C, under a 5% CO_2_ atmosphere. To measure the membrane potential, the cells were stained with 100 nM DiBAC_4_(3) (Life Technologies, Eugene, OR, USA) for 10 min at 37°C. The samples were then put on ice and the mean fluorescence intensity of the probe was detected using a BD FACSCanto Flow Cytometer (BD Biosciences, San Jose, CA, USA) with a 488-nm laser for excitation and a 530/30 nm emission filter. All flow cytometric analyses were done using FlowJo vX software (TreeStar Inc. OR, USA). As a positive control, neutrophils were exposed to graded K^+^ concentrations (1, 5, 10, 25, 38, 50, 75, and 100 mM).

### Antimicrobial Peptides/Proteins, Mucins, and Bacterial Killing

Subcultures of PAO1 were adjusted to 2.5 × 10^5^ bacteria/ml in 10 mM phosphate buffer (pH 7.4) and incubated with 0–4 mg/ml CFAM or salivary mucins for 20 min at RT. Each sample was then incubated for 2 h at 37°C with gentle agitation, either alone or with one of the following antimicrobial peptide/proteins preparations: 6 μg/ml of recombinant human neutrophil lysozyme; lysozyme and 0–5 mg/ml of chondroitin sulfate; 4 μg/ml of human β defensin-2 (HBD-2); or neutrophil granule extract containing 8 μg/ml of lysozyme (Sigma-Aldrich). The samples were then diluted in PBS, plated on Müller-Hinton agar, incubated overnight at 34°C, and CFU were counted by standard plate-counting procedures (Bals et al., 1998; Felgentreff et al., 2006).

### Statistical Analysis

The results are presented as mean ± SEM. Data with multiple group comparisons were analyzed using ANOVA and data with two group comparisons were analyzed with the Student’s *t*-test (Prism v8.0, Graph Pad Software Inc., San Diego, CA).

### Ethics Review and Informed Consent

Patients were recruited from the CF clinic for adults at the CIUSSS-CHUS. Ethics approval was obtained from the Centre de Recherche Clinique du CHUS Institutional Review Board for sputum, saliva, and blood procurement in all CF subjects and healthy volunteers that participated in the study. All participants provided informed consent prior to inclusion into the study.

## Results

### Mucins Protect *Bcc* and *P. aeruginosa* Against Neutrophil Killing

**Figure 1:**
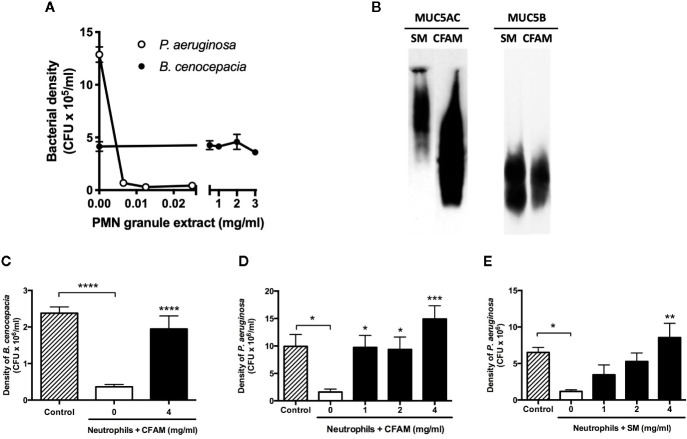
Bacterial killing by neutrophil granule extracts and neutrophils with and without mucins. **(A)** Human neutrophil granule extracts markedly inhibited *Pseudomonas aeruginosa* (PAO1) growth but did not affect *Burkholderia cenocepacia* (C5424) density (n = 3). **(B)** Characterization of CF airway mucins (CFAM) and salivary mucins of healthy volunteers (SM). Samples of CFAM (200 μg) and SM (200 μg) were separated on a 0.7% agarose gel. The proteins were transferred to nitrocellulose membranes and revealed with either goat polyclonal mucin MUC5B IgG or MUC5AC concentrated Clone B442 antibody. **(C)** Neutrophil killing of *B. cenocepacia* (n = 9) and **(D)** *P. aeruginosa* (n = 9) in the absence or presence of CFAM or **(E)** SM (n = 7). CFAM and SM suppressed neutrophil-mediated killing of PAO1 and C5424 bacteria after incubation for 2 h at 37°C. (n = 9, *P < 0.05, **P < 0.01, ***P < 0.001, ****P < 0.0001).

While less than 10 µg/ml of neutrophil granule extract completely inhibited the growth of *P. aeruginosa* (n = 3, p < 0.0001 vs. control), a 300-fold higher concentration of the neutrophil granule extract did not affect *B. cenocepacia* growth (**Figure 1A**). Mucins purified from either saliva or CFAM reacted with antibodies specific to both Muc5AC and Muc5B (**Figure 1B**). Despite their markedly different susceptibilities to non-oxidative killing, the growth of both *B. cenocepacia* and *P. aeruginosa* was readily counteracted by neutrophils; in the presence of CFAM, however, bacterial killing of either strain by neutrophils was strongly inhibited (**Figures 1C, D**). A protection against neutrophil killing was also observed when salivary mucins from healthy volunteers was used instead of CFAM (**Figure 1E**).

### CF Airway Mucins and Neutrophil Phagocytosis

**Figure 2:**
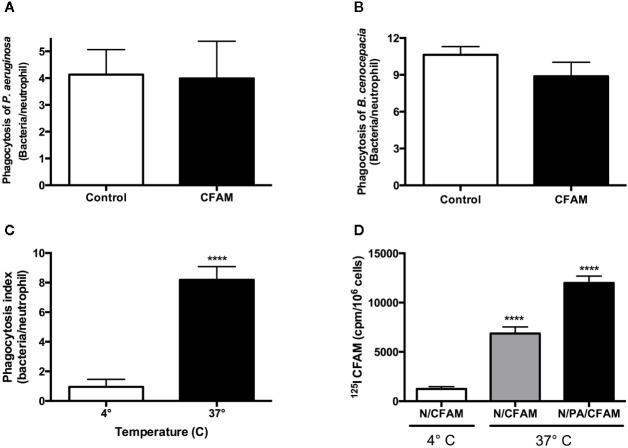
Effect of CFAM on neutrophil-mediated bacterial phagocytosis and mucin adherence to resting and activated neutrophils. *Pseudomonas aeruginosa* (PAO1) and *Burkholderia cenocepacia* (C5424) bacteria were incubated 20 min at RT without (control) and with CFAM and added to human neutrophils in a ratio of 10 bacteria to 1 neutrophil for 1 h at 37°C. The phagocytosis indices for neutrophils exposed to **(A)** *Pseudomonas aeruginosa* (n = 6) and **(B)** *Burkholderia cenocepacia* (n = 5) were not different in the presence of 4 mg/ml of CFAM (p > 0.05). Opsonized *Pseudomonas aeruginosa* were incubated in the presence of 4 mg/ml CFAM and blood-derived neutrophils at 37 or 4°C for 1 h to determine the effect of temperature on **(C)** phagocytosis (n = 3), and on **(D)** ^125^I-CFAM adherence or uptake by neutrophils (n = 3), determined by measuring the radioactivity of cell pellets after extensive washing. N/CFAM: neutrophils with CFAM; N/PA/CFAM: neutrophils with *Pseudomonas aeruginosa* and CFAM. (****P < 0.0001 vs. 4°C).

The number of *P. aeruginosa* or *B. cenocepacia* bacteria in phagocytic compartments was similar following treatment with or without 4 mg/ml CFAM (**Figures 2A, B**). Phagocytosis of opsonized *P. aeruginosa* incubated with CFAM was robust at 37°C but was decreased 8-fold at 4°C (**Figure 2C**), indicating that the bacteria observed at 37°C had been ingested by the neutrophils. Electron microscopy confirmed that the bacteria were truly ingested and not merely adherent to the neutrophil surface (data not shown). Furthermore, the uptake of ^125^I-labeled CFAM by neutrophils was increased at 37°C particularly during phagocytosis (**Figure 2D**), indicating that CFAM uptake by neutrophils is an energy-dependent process that is enhanced during bacterial phagocytosis.

### Mucins Suppress the Neutrophil Oxidative Burst

**Figure 3:**
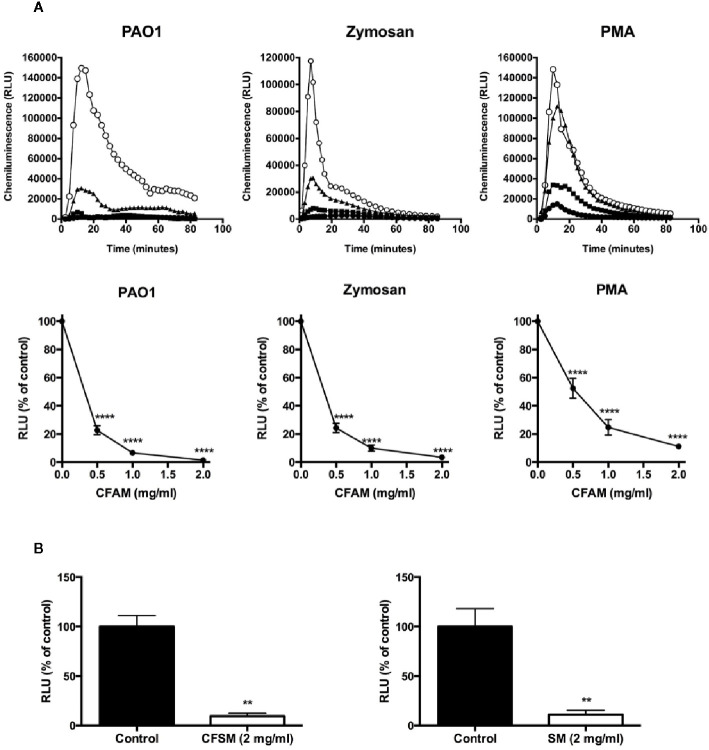
Mucin suppression of intracellular chemiluminescence in neutrophils activated by opsonized PAO1 bacteria, opsonized zymosan and PMA. **(A)**. Neutrophils isolated from the peripheral blood of healthy volunteers were incubated with opsonized PAO1 at a bacteria:neutrophil ratio of 2:1, 2.5 mg/ml of opsonized zymosan, or 1 µg/ml of PMA. Luminol-enhanced chemiluminescence in the presence of superoxide dismutase and catalase was measured over time in a luminometer. (Top row: open circles = no CFAM; triangles = CFAM, 0.5 mg/ml; squares = CFAM, 1 mg/ml; closed circles = CFAM, 2 mg/ml). The bottom row shows the mean ± sem of the maximal RFU data points recorded in repeated experiments. (n = 4 experiments, ****P < 0.001 vs. no mucin). **(B)** Mucins derived from the saliva of CF (CFSM) and non-CF (SM) individuals also suppressed neutrophil chemiluminescence in the presence of opsonized PAO1 bacteria (n = 3 experiments, **p < 0.01 vs. non mucin).

Exposure of neutrophils to opsonized *P. aeruginosa*, opsonized zymosan, or PMA induced a robust oxidative burst as detected by chemiluminescence (**Figure 3A**). CFAM induced a concentration-dependent suppression of the neutrophil oxidative burst stimulated by bacterial phagocytosis, opsonized zymosan or PMA. The neutrophil oxidative burst was also suppressed by mucins purified from CF saliva and from healthy volunteers (**Figure 3B**).

### Effect of Charge Neutralization on Neutrophil Oxidant Synthesis

**Figure 4:**
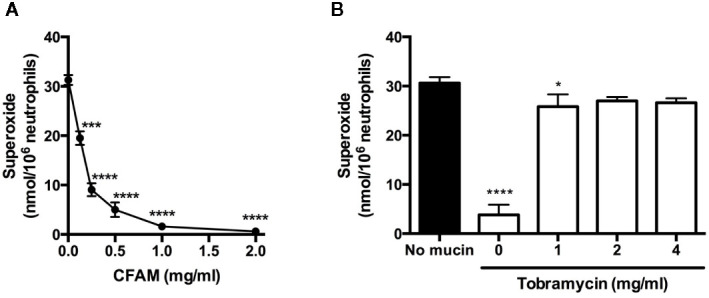
Effects of mucin alone, and mucin with tobramycin on neutrophil superoxide release. **(A)** Neutrophils from healthy volunteers were stimulated with opsonized zymosan and superoxide release was determined by measuring the reduction of ferricytochrome *c* in the presence of cystic fibrosis airway mucin. **(B)** The cationic aminoglycoside, tobramycin was added to neutrophils stimulated with opsonized zymosan in the presence of 2 mg/ml of CFAM (white columns), and superoxide release was determined with ferricytochrome *c*. (n = 3, *p < 0.05, ***p < 0.001, ****p < 0.0001 vs. no mucin).

Because CF airway mucins have a strong negative charge, we next determined whether neutralization of the charge with the cationic aminoglycoside tobramycin could restore the neutrophil oxidative burst in the presence of CFAM. As with luminol-dependent chemiluminescence, CFAM caused a concentration-dependent decrease in superoxide release from neutrophils stimulated with opsonized zymosan as measured with ferricytochrome *c* reduction (**Figure 4A**). The addition of 1–4 mg/ml of tobramycin to the neutrophils stimulated with opsonized zymosan markedly reversed the suppression of superoxide release induced by 2 mg/ml of CFAM (**Figure 4B**).

### Mucins Depolarize Neutrophil Membranes

**Figure 5:**
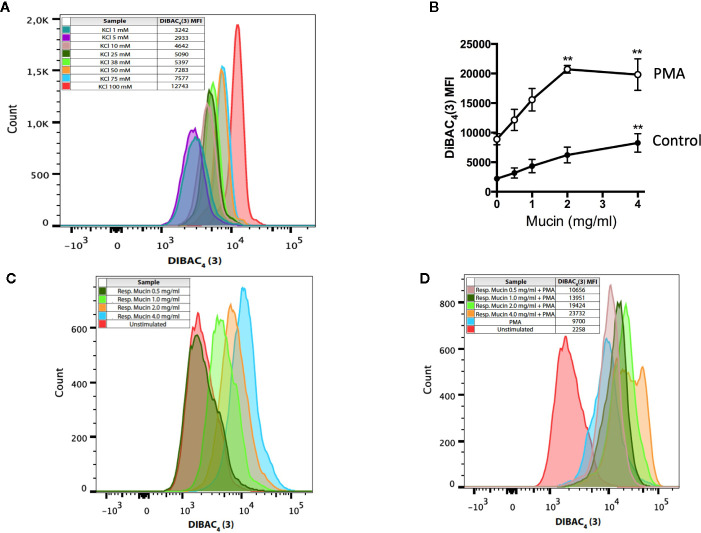
Respiratory mucins promote and potentiate PMA-induced neutrophil membrane depolarization. **(A)** Neutrophils were exposed to graded K^+^ concentrations. Membrane depolarization is indicated by the shift in the intensity of DiBAC4(3) fluorescence. Graded potassium solutions were made by increasing the KCl concentration in the solution. For graded K^+^ solutions, the KCl concentration was set to 1, 5, 10, 25, 38, 50, 75, and 100 mM. **(B)** Human neutrophils were incubated in the presence of respiratory mucins (•, 0.5–4 mg/ml) or stimulated with 200 nM PMA in the presence of respiratory mucins (<). After a 20-min incubation, the cells were stained with 100 nM DiBAC_4_(3). The transmembrane potential was determined by flow cytometry as described in Materials and Methods and is presented as mean fluorescence intensity (MFI) of the DiBAC_4_(3) probe. Means ± s.e.m. of four independent experiments are shown. **(C)** Representative flow cytometric histograms showing a concentration-dependent increase in DiBAC4(3) fluorescence in resting neutrophils incubated with mucins, indicating plasma membrane depolarization. **(D)** Exposure of neutrophils to increasing concentrations of respiratory mucins potentiated membrane depolarization in PMA-stimulated neutrophils. The data are representative of four separate experiments in four donors. (**p < 0.01 vs. no mucin).

Neutrophil plasma membrane potential can be determined using the lipophilic anion DiBAC_4_(3), a plasma-membrane selective dye that tracks increases in membrane potential of neutrophils as is induced by extracellular KCl (**Figure 5A**). Resting neutrophils have a membrane potential of -58 mV (Jankowski and Grinstein, 1999). The addition of CFAM alone to resting neutrophils caused a concentration-dependent increase in membrane depolarization (**Figures 5B, C**), consistent with previous reports indicating that anionic polymers alone can depolarize cell membrane potential (Hagenfeld et al., 2010). As expected, PMA alone increased membrane potential, in keeping with previous reports (Jankowski and Grinstein, 1999). However, the addition of mucin to PMA-stimulated neutrophils acted synergistically to markedly depolarize the plasma membrane, as evidenced by increases in the DiBAC_4_(3) mean fluorescence index (MFI) to levels much higher than those recorded with 100 mM KCl or PMA alone (**Figures 5B, D**).

### Mucins Protect Bacteria Against Non-Oxidative Killing by Cationic Peptides and Proteins

**Figure 6:**
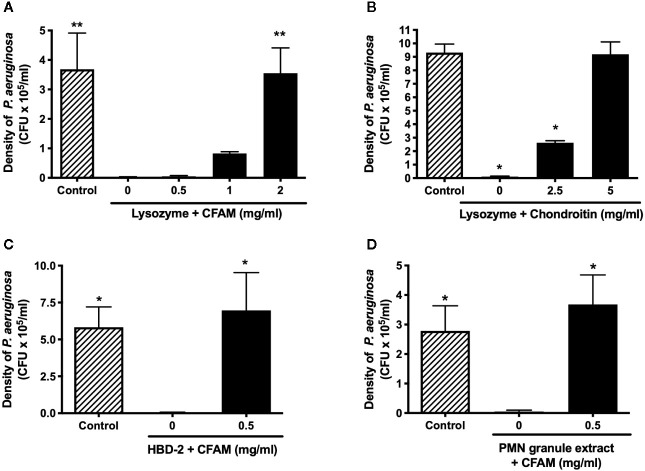
Effect of mucins on non-oxidative killing by cationic peptides and proteins. **(A)** Recombinant human neutrophil lysozyme (6 µg/ml) effectively killed *P. aeruginosa* bacteria and **(B)** the addition of chondroitin to lysozyme protected bacteria (n = 4). Airway mucins purified from CF mucus also protected *P. aeruginosa* against killing by **(C)** 4 µg/ml of HBD-2 and **(D)** neutrophil granule extracts containing 8 µg/ml of lysozyme (n = 4 (*p < 0.05, **p < 0.01).

Human recombinant neutrophil lysozyme was found to eradicate *P. aeruginosa* but this effect was reversed in the presence of CFAM, in a concentration-dependent manner (**Figure 6A**). We next determined whether anionic polymeric glycans other than mucins also protect against lysozyme-mediated bacterial killing. As shown in **Figure 6B**, co-incubation lysozyme with chondroitin, an anionic glycosaminoglycan, completely protected the bacteria against killing. Finally, CFAM was found to protect *P. aeruginosa* bacteria against other cationic peptides and proteins, including HBD-2 and PMN granule extract (**Figures 6C, D**).

## Discussion

Despite the large number of neutrophils in the airways, chronic bacterial infection is a prominent feature of CF lung disease and is associated with a steady decline in lung function. A marked reduction in mucus clearance from the airways (mucostasis) is a hallmark CF lung disease (Esther et al., 2019). Mucostasis is also observed in individuals with chronic obstructive pulmonary disease or COPD (Henderson et al., 2014; Kesimer et al., 2017; Hill et al., 2018) and correlates with disease severity (Kesimer et al., 2017). These considerations led us to hypothesize that there is a link between mucins and neutrophil dysfunction. We now report that mucins, at concentrations found in the airways of patients with mucostasis markedly suppress both oxidative and non-oxidative bactericidal effects of neutrophils. Mucins derived from three different sources, CF sputum, CF saliva, and the saliva of healthy volunteers all suppressed the neutrophil oxidative burst.

The NADPH oxidase complex (NOX2) is comprised of several protein subunits assembled at the neutrophil membrane (or at the phagolysosome membrane) following stimulation (Dinauer, 2005). Activated NADPH oxidase transfers a large number of electrons derived from NADPH in the cytosol to the extracellular milieu, including the phagosome, where the electrons reduce oxygen to superoxide. The membrane potential of resting neutrophils is approximately -58 mV (Jankowski and Grinstein, 1999). During an oxidative burst, electron efflux creates a current that depolarizes the neutrophil membrane at a rate that would attain 11 kV min^-1^ if it were not for charge compensation by the flow of NADPH-derived protons through the Hv1 proton channel (DeCoursey and Hosler, 2014). Proton efflux limits the membrane depolarization of PMA-stimulated neutrophils to approximately +58 mV (Jankowski and Grinstein, 1999). Plasma membrane depolarization to levels above +60 mV is associated with inhibition of NADPH oxidase activity. The addition of mucin to resting or activated neutrophils results in depolarization of the trans-membrane potential and likely contributes to decreasing the neutrophil’s capacity to reduce oxygen to superoxide.

Neutrophils with an NADPH oxidase deficiency can readily kill catalase-negative bacteria, but not catalase-positive organisms. Pathogens in both CF and CGD are most often catalase-positive organisms. Bcc are catalase-positive bacteria and are highly resistant to non-oxidative killing (Speert et al., 1994). Neutrophils require an intact oxidative mechanism to kill this rare pathogen observed almost exclusively in patients with either CGD or CF (Vethanayagam et al., 2011). Furthermore, most of the strains of *Streptococcus anginosus* reported to infect patients with CGD (Falcone and Holland, 2013) can also cause respiratory exacerbations in CF (Grinwis et al., 2010) and are low H_2_O_2_ producers, consistent with the concept that neutrophils in the CF airway environment may have a functional NADPH oxidase deficiency associated with mucostasis.

Inflammatory abnormalities are present in patients with both CF and CGD. The role of NADPH oxidase in the clearance of neutrophils and the resolution of inflammation is of particular interest. NADPH oxidase is essential for phosphatidylserine oxidation and expression on the outer leaflet of the plasma membrane, a key process initiating apoptosis (Hampton et al., 2002). If PS is not externalized, then the neutrophil is not recognized by PS-receptors on macrophages and programmed neutrophil clearance known as efferocytosis fails. Efferocytosis has been shown to be deficient in patients with NADPH oxidase deficiency, and contributes to the exaggerated inflammation characteristic of CGD (Sanmun et al., 2009). Failure of sputum neutrophils to undergo apoptosis as determined by deficient PS expression on the outer membrane leaflet is also associated with more severe CF lung disease (Houston et al., 2013). The similarities in the exaggerated inflammation of CF and CGD support the concept that abnormalities in NADPH oxidase function may underlie at least some mechanisms relevant to both diseases.

Mucin concentration and clearance from the airways are regulated by CFTR. It is conceivable that a transient decrease in CFTR activity represents a physiological adaptive response of airway cells against neutrophil-derived oxidants to dampen NADPH oxidase-dependent release of ROS and limit mucosal exposure to oxidants. Consistent with this hypothesis, the CFTR function of airway epithelial cells is transiently suppressed by oxidant exposure (Cantin et al., 2006a; Cantin et al., 2006b), and recovery of CFTR function occurs within 4 h after exposure (Clunes et al., 2012). In the lung, recovery of CFTR function would be expected to increase airway surface liquid hydration and clear mucus from the bronchi, thus restoring an environment that is favorable for NADPH oxidase function. In contrast, individuals with sustained CFTR dysfunction such as in CF and possibly COPD (Dransfield et al., 2013) are at risk of prolonged NADPH oxidase inhibition, thus preventing optimal killing of pathogens and resolution of inflammation, as observed herein in the case of neutrophils incubated with CFAM.

Several anti-bacterial mechanisms not related to oxidants are defective in the CF airway, and likely increase susceptibility to *P. aeruginosa* infection (Pezzulo et al., 2012). Accordingly, we observed that mucins inhibit killing of *P. aeruginosa* by neutrophil granule extracts, neutrophil lysozyme and HBD-2. Neutrophil granules contain peptides and proteins that kill bacteria, mainly through mechanisms dependent upon their positive charge. The importance of these pathways in antibacterial host defense has been confirmed in several studies using transgenic mice (Belaaouaj et al., 1998; Beaumont et al., 2014). Despite retaining a normal neutrophil oxidative burst capacity, these mice are highly susceptible to lethal bacterial infections. (Belaaouaj et al., 1998; Tkalcevic et al., 2000; Reeves et al., 2002; Ahluwalia et al., 2004). Further evidence of the importance of non-oxidative antibacterial host defenses stems from the investigation of neutrophil extracellular trap (NET) formation. NETs indeed feature bound cationic proteins (including elastase, cathepsin G, and lactoferrin) that participate in the killing of Gram-positive and -negative bacteria (Brinkmann et al., 2004). Our results indicate that CFAM markedly inhibits the bactericidal properties of neutrophil granule extracts and that of key cationic antimicrobial molecules. These effects of CFAM are likely related to charge neutralization of cationic antimicrobial agents since other anionic polymeric glycoproteins have been shown to suppress the antibacterial properties of neutrophil-derived cathelicidin LL-37, β -defensins, and lactoferrin (Weiner et al., 2003; Felgentreff et al., 2006; Baranska-Rybak et al., 2006).

Collectively, our observations point to potential therapeutic targets for CF. Clearly, the most exciting therapeutic target in CF is CFTR itself. Correction of CFTR is expected to lead to appropriate regulation of mucin hydration and mucus clearance, thus preventing the sustained concentration and stasis of mucins at the airway surface. A second therapeutic target is the abundance and viscoelastic properties of mucins in the CF airways. In particular, the expression and viscoelastic properties of MUC5AC were reported to be critically affected by oxidative stress and neutrophil elastase (Takeyama et al., 2000; Shao and Nadel, 2005; Yuan et al., 2015). These properties may be amenable to therapies involving anti-inflammatory, antioxidant and antiprotease molecules. Treating mucostasis in CF infants and adults with hypertonic saline and dornase alfa has shown clinical benefit and pre-clinical studies of agents that disrupt disulfide bonds in mucins are promising (McCoy et al., 1996; Donaldson et al., 2006; Ehre et al., 2019). Finally, the current study raises the possibility that cationic antibiotic therapy with tobramycin can restore neutrophil oxidative burst in the presence of mucins. Other cationic molecules with antibiotic and anti-inflammatory potential, such as lysozyme, lactoferrin, secretory leukocyte protease inhibitor (SLPI), and elafin, may also be of interest for therapeutic development in CF.

## Data Availability Statement

The datasets presented in this article are not readily available because: No restrictions. Requests to access the datasets should be directed to andre.cantin@usherbrooke.ca.

## Ethics Statement

The studies involving human participants were reviewed and approved by Comité d’Éthique de la Recherche, CIUSSS Estrie–Centre Hospitalier Universitaire de Sherbrooke. The patients/participants provided their written informed consent to participate in this study.

## Author Contributions

AMC conceived the project, planned experiments and wrote the manuscript. CO performed the assays and wrote and revised the manuscript. AC conducted the experiments of neutrophil transmembrane potential difference and revised the manuscript. PM provided advice for neutrophil purification and characterization and revised the manuscript.

## Funding

This work was supported by a grant in aid of research from Cystic Fibrosis Canada (grant number 2409). AC and PM are members of the FRQS-funded Centre de Recherche Clinique du CHUS.

## Conflict of Interest

The authors declare that the research was conducted in the absence of any commercial or financial relationships that could be construed as a potential conflict of interest.
